# Whole-Genome Sequences of Four *Corynebacterium* CDC Group F-1 Strains Isolated from Urine

**DOI:** 10.1128/genomeA.01537-16

**Published:** 2017-02-02

**Authors:** Anne-Marie Bernier, Geoffrey A. Peters, Kathryn Bernard

**Affiliations:** a Department of Biology, Université de Saint-Boniface, Winnipeg, Manitoba, Canada; b National Microbiology Laboratory (NML), CSCHAH Site, Public Health Agency of Canada, Winnipeg, Manitoba, Canada; c Department of Medical Microbiology, University of Manitoba, Winnipeg, Manitoba, Canada

## Abstract

Three draft and one complete genome sequence from strains isolated from urine and consistent with *Corynebacterium* CDC group F-1 were assembled and studied. Genome sizes ranged between 2.3 and 2.44 Mb, with G+C content between 60.4% and 60.7%.

## GENOME ANNOUNCEMENT

In 1981, the Centers for Disease Control (CDC) published an identification scheme for isolates consistent with the genus *Corynebacterium* based on biochemical results and other phenotypic traits (1). Some taxon groups which did not precisely fit with existing species definitions were assigned interim “CDC group” names. Most have been subsequently assigned to *Corynebacterium* species or to other genera, except those consistent with *Corynebacterium* CDC group F-1 (2). Here, four isolates (NML identifiers 98-0116, 120713, 130628, and 140438, all from urine) that were lipophilic, produced urease, and fermented glucose, maltose, and sucrose but were variable for nitrate reduction were found to have ≥98.8% identity by 16S rRNA gene sequencing to CDC group F-1 strains G5911 and G4330 (GenBank accession numbers X81904 and X81905, respectively). These isolates were studied by whole-genome sequencing to establish if they represented a single or several phylogenetic groups.

DNA was extracted using the TruSeq DNA high-throughput (HT) sample preparation kit. Paired-end whole-genome shotgun libraries were constructed using TruSeq Nano DNA HT library preparation kit. A mate-pair library for strain NML 98-0116 was prepared using the Nextera mate-sample prep kit (Illumina). Sequencing was performed using a MiSeq sequencer (Illumina), and assembly was performed with SPAdes (version 3.1.1) (Table 1). Contigs from strain NML 98-0116 were further assembled into 19 scaffolds using mate-pair reads with SSPACE version 2.0.0. Overlapping ends of the scaffolds and four repeat nodes were then identified and joined using Gap4 from the Staden software package to produce a single chromosomal contig of 2.36 Mb (3).

**TABLE 1:** Genome sequencing statistics and accession numbers

Strain	Genome size (bp)	No. of contigs (>1 kb)	G+C content (%)	No. of genes	Genome coverage (×)	Accession no.
NML98-0116	2,365,509	1	60.7	2,220	37	CP017639
NML120713	2,445,057	30	60.7	2,327	61	MLCR00000000
NML130628	2,304,732	45	60.4	2,166	143	MLAL00000000
NML140438	2,333,167	31	60.6	2,253	103	MLCQ00000000

Genomes were annotated using the National Center for Biotechnology Information (NCBI) Prokaryotic Genome Annotation Pipeline (PGAP) (https://www.ncbi.nlm.nih.gov/genome/annotation_prok). Assembled whole genomes demonstrated essentially identical G+C contents, genome sizes, and number of coding regions (Table 1). The genomes were compared to each other using JSpeciesWS to calculate the average nucleotide identity values using BlastN (ANIb) (4). Using this approach, NML 98-0116 and NML 120713 had ANIb scores greater than 96% to each other, suggestive of being members of the same species. Strains NML 130628 and NML 140438, however, did not have ANIb scores higher than 90.5% to each other nor to the other strains, indicating that these may represent two new separate species and that this group was heterogenic. Strains were analyzed for prophage sequences using PHASTER (5). NML 140438 was found to harbor an intact prophage, the *Rhodococcus* phage ComicSans, consisting of 32.2 kb and 22 coding sequences (CDSs), with a G+C content of 56.72% (5). The genome of each strain lacked a gene encoding fatty acid synthase (FAS), as expected for lipophilic *Corynebacterium* species (6). NML 98-0116, NML 120713, and NML 140438 had an *ermX* gene (associated with resistance to erythromycin and clindamycin) homologous to that found in *Corynebacterium urealyticum*, *Corynebacterium jeikeium*, and *Corynebacterium striatum*. This was consistent with published antibiograms for these strains (7). The genomes of all four strains contained homologs to genes known to be involved in the synthesis of mycolic acids (8), consistent with the expectation for the detection of cell wall mycolates (9).

### Accession number(s).

Complete or draft genome sequences of four *Corynebacterium* CDC group F-1 isolates have been deposited in GenBank under accession numbers shown in Table 1.
